# Patient Perceptions and Satisfaction With Virtual Clinics During the COVID-19 Pandemic: A Cross-Sectional Study

**DOI:** 10.7759/cureus.42450

**Published:** 2023-07-25

**Authors:** AlHanouf M AlJaloud, Abdulaziz Al Suwyed, Khalid H Al Zoman, Mohammad Y Tabbaa, Asirvatham Alwin Robert, Abeer M Al‐Nowaiser, Faisal Alotaibi, Mohammed A Alfaifi, Sultan A Almubarak

**Affiliations:** 1 Plastic Surgery, King Fahad Medical City, Riyadh, SAU; 2 Dentistry, King Saud Bin Abdulaziz University for Health Sciences, Riyadh, SAU; 3 Dentistry, King Faisal Specialist Hospital and Research Centre, Riyadh, SAU; 4 Endocrinology and Diabetes, Prince Sultan Military Medical City, Riyadh, SAU; 5 Pediatric Dentistry, King Abdulaziz University, Jeddah, SAU; 6 Neurological Surgery, King Faisal Specialist Hospital and Research Centre, Riyadh, SAU; 7 Emergency Medicine, King Faisal Specialist Hospital and Research Centre, Riyadh, SAU; 8 Innovation and Knowledge Translation, Saudi National Institute of Health, Riyadh, SAU

**Keywords:** kingdom of saudi arabia (ksa), a cross-sectional study, covid-19, telemedicine services, virtual clinics

## Abstract

Background

Virtual clinics played an important role for many patients during the COVID-19 pandemic. We conducted this cross-sectional study to evaluate patient perceptions and their satisfaction with virtual clinics during and after COVID-19 in Saudi Arabia.

Methods

An online questionnaire-based survey with questions in both Arabic and English was conducted among patients who attended outpatient clinics at King Faisal Specialist Hospital and Research Center, Riyadh, Saudi Arabia from May 2021 to September 2021. Demographic variables, the clinic type, and the level of satisfaction with the remote appointments were recorded. Descriptive statistics and logistic regression analysis were used to analyze the data.

Results

A total of 1274 participants filled out the survey. Of them, 831 (65.23%) were females, and 749 (58.79%) were aged 18 to 30 years old. Of the sample studied, 411 (32.26%) had appointments with their healthcare provider remotely since the beginning of the pandemic; 311 (75.67%) were satisfied or highly satisfied with the remote appointments; and 198 (48.18%) participants desired to continue using virtual services post-COVID-19 pandemic. Logistic regression analysis showed that females were more satisfied with virtual clinics than males (OR= 1.18, 95% CI (1.01, 1.40), p=0.04). The age group of 18 to 30 was more satisfied than other age groups (OR= 53.23, 95% CI (2.01, 1347.18), p=0.02).

Conclusion

The majority of the participants who used virtual clinics were satisfied with the service. Nearly half of the participants wanted to continue using virtual services even after the COVID-19 pandemic was over. More effort should be made to increase patient awareness and knowledge about virtual clinics.

## Introduction

On 11th March 2020, the World Health Organization (WHO) declared COVID-19 a global pandemic [1]. Severe acute respiratory syndrome coronavirus 2 (SARS-CoV-2) spreads primarily through respiratory droplets and aerosols, in addition to surface contamination. The risk of contracting COVID-19 is heightened by close contact with an infected individual at a distance of less than 1 m [2]. Due to the high contagion rate of SARS-CoV-2, new models of healthcare delivery were explored to avoid face-to-face consultations between clinicians and patients. This significantly reduced the risk of COVID-19 transmission [3-5].

During the COVID-19 pandemic, national lockdowns, cancellations, and delays of various procedures, as well as the unavailability of healthcare professionals due to the fear of viral contamination, resulted in the disruption of health services in both developed and developing countries [6]. Furthermore, outpatient visits posed a significant health risk for patients and physicians as the virus spread rapidly. Therefore, it was essential to divert patients from inpatient care to prevent overwhelming healthcare services. Telemedicine, which uses telecommunication technologies, is a suitable method of healthcare delivery. According to WHO, telemedicine involves using information and communication technologies for the diagnosis, treatment, and prevention of disease, as well as research and education in the health field [7]. It is a secure process that takes place not only between the patient and the specialist but also between the healthcare providers [8,9]. Remote consultations help reduce disease transmission by encouraging patients to reduce their physical visits to the hospital and primary clinics enabling clinicians to work remotely [10,11].

There is a significant shortage of studies that assess patient perceptions of receiving medical care virtually, as well as its advantages and challenges. Therefore, we conducted the present study to evaluate patient satisfaction and response towards receiving routine medical care virtually. We also investigated the association between socio-demographic characteristics and patient satisfaction during and after the COVID-19 pandemic in Saudi Arabia.

## Materials and methods

Study design and setting

This cross-sectional survey was conducted at the King Faisal Specialist Hospital and Research Centre in Riyadh, Saudi Arabia, from May 2021 to September 2021. The study was conducted in the outpatient clinics of various departments at the hospital.

Sample size calculation

The research team estimated the required sample size based on the formula n = z^2 * p * (1 - p) / e^2. Here, "n" is the required sample size, "p" is the prevalence of patients attending outpatient clinics, q is 1−p (1−0.373) = 0.627, z = 1.96 at 95% confidence interval (CI), and e = 5% margin of error. Applying the values to the above formula and considering the inclusion of three regions in Saudi Arabia, the minimum required sample size for this study was 1260 patients.

Inclusion and exclusion criteria

To be eligible for participation in the study, the participants needed to satisfy the following criteria: be 18 to 90 years of age and Saudi citizens or residents. Patients who were not willing to participate or did not have access to an internet connection were excluded from the present study.

Ethical consideration

The study was conducted in accordance with the Declaration of Helsinki principles. The Research Ethics Committee at King Faisal Specialist Hospital and Research Centre, Riyadh, Saudi Arabia, approved the study protocol. Informed consent was obtained from each participant before they proceeded to fill out the data collection form.

Data collection procedure

An online questionnaire was used to collect data on the participants' acceptance of the virtual clinic concept during and after COVID-19. The questionnaire involved 26 questions and was divided into four parts: demographic variables, type of hospital and clinic visit, questions about COVID-19, and remote appointments. The survey was conducted using SurveyMonkey® (SurveyMonkey Inc., San Mateo, CA, USA), and all data was protected by Secure Sockets Layer (Netscape Communications Corp., Mountain View, CA, USA) encryption. The survey used a skip logic pattern and included both closed-ended and open-ended questions [12,13]. The questionnaire was distributed via social media platforms, primarily WhatsApp (Meta, Menlo Park, CA, USA). The convenience snowball sampling method was utilized for participant recruitment. The decision to distribute the questionnaire through online channels was deemed appropriate due to the research objectives and the constraints imposed by COVID-19 pandemic restrictions during the data collection phase, which limited hospital access to emergency situations only.

Statistical analysis

Descriptive analysis was used to analyze the data, and frequencies and percentages were used to express the results. Mann-Whitney and Kruskal Wallis tests were used to compare non-normally distributed data. A logistic regression analysis was performed to recognize the independent variables associated with patient satisfaction. A p-value of less than 0.05 was considered statistically significant. Data analysis was carried out using SPSS Statistics version 26 (IBM Corp., Armonk, NY, USA).

## Results

A total of 1274 participants responded to the questionnaire. The majority of respondents were in the 18 to 30 age group (n=749, 58.79%), followed by the 31 to 40 age group (n=194, 15.23%). Most respondents were females (n=831, 65.23%) and college-educated (n=723, 56.75%), and almost all were Saudi nationals (n=1221, 95.84%). Government hospitals were the most commonly used healthcare facilities (n=737, 57.85%), followed by private hospitals (n=694, 54.24%). The majority of participants reported visiting primary healthcare facilities only when they had a health problem (n=990, 77.71%). Regarding specialized clinics, 411 participants (32.26%) reported visiting dental clinics, and 114 visited diabetes and endocrine clinics (8.95%). Table 1 displays the demographic characteristics and survey responses of the study participants.

**Table 1:** Frequency (n) and percentage (%) of responses to the questionnaire MOH: Ministry of Health

Sample characteristics	Number (n=1274)	Percentage
Age groups		
18 – 30	749	58.79
31 – 40	194	15.23
41 – 50	147	11.54
51 – 60	109	8.56
61 – 70	40	3.14
> 70	17	1.33
Gender		
Male	430	33.75
Female	831	65.23
Sex ratio, M:F	0.52:1	
Level of education		
Below college	413	32.42
College	723	56.75
Postgraduate	128	10.05
Where are you working?		
Not working	524	41.13
Private sector	187	14.68
Government sector	303	23.78
Retired	101	7.93
Others	150	11.77
What type of healthcare facility do you use?		
Government hospitals	737	57.85
Private hospitals	694	54.24
MOH primary health center	211	16.56
Private polyclinics	252	19.78
Why do you visit your primary healthcare facility?		
For regular check-ups	344	27.00
Refill my medication	169	13.27
Only if I have a problem	990	77.71
Others	67	5.26
Which primary clinic do you visit? Choose all that apply		
Dental clinic	411	32.26
Diabetes and endocrine clinic	114	8.95
ENT clinic	72	5.65
Family medicine clinic	177	13.89
Dermatology centers	71	5.57
How often do you visit the healthcare facility annually?		
1 – 3	743	58.32
4 – 6	353	27.71
7 – 10	125	9.81
>10	49	3.85
Do you have to travel to get to your healthcare facility?		
Yes	111	8.72
No	1157	90.82
Have you been tested for COVID-19?		
Yes, and it was positive (infected)	154	12.09
Yes, and it was negative (not infected)	593	46.55
Yes, and the results were sometimes positive and sometimes negative	35	2.75
No	488	38.30
How many times have you taken a COVID-19 test?		
1	364	28.57
2	147	11.54
3	92	7.22
4	41	3.22
>5	140	10.99
Do you fear visiting the healthcare facility during the COVID-19 pandemic?		
Yes	564	44.27
No	700	54.95
Do you take precautions during your visit to the healthcare facility?		
Yes	912	71.59
No	355	27.86
Has the current pandemic affected your access to medical treatment?		
No, everything is running as usual	729	57.22
I decided to cancel/postpone my appointments	282	22.14
The healthcare facility canceled/postponed my appointments	236	18.52
Have you had any appointments related to your disease with your healthcare provider remotely since the beginning of the COVID-19 pandemic?		
Yes	411	32.26
No	821	64.44
If you have had a remote appointment with your healthcare provider since the start of the pandemic, how satisfied are you with the provided service?		
Highly satisfied	184	44.77
Satisfied	127	30.90
Neither satisfied nor dissatisfied	54	13.13
Dissatisfied	23	5.60
Highly dissatisfied	23	5.60
If you have had a remote appointment with your healthcare provider since the start of the pandemic, would you like to continue the same way?		
Yes	198	48.18
No	213	51.82
How was your remote appointment conducted with your healthcare provider?		
Telephone, cellphone	202	49.15
Through social networking applications (WhatsApp, etc.)	61	14.84
Video calls (Skype/Zoom audiovisual)	59	14.36
MOH phone services via 937	65	15.81
Others	24	5.84
How likely are you to consider remote appointments instead of in-person appointments after the pandemic?		
Very likely	147	11.54
Likely	106	8.32
Neither likely nor unlikely	101	7.93
Unlikely	72	5.65
Very unlikely	52	4.08
Why do you prefer remote appointments?		
I found out that the remote medical service is better for me	57	13.87
Saves my time	175	42.58
Less costly	56	13.63
More convenient than my regular visits	77	18.73
To avoid the traffic	46	11.19

Of the study population, 743 (58.32%) visited the healthcare facility one to three times annually, while 353 (27.71%) visited four to six times per year. The majority of responders (n=1157, 90.82%) did not need to travel to reach the healthcare facility. And 557 (43.72%) mentioned that the travel cost was less than 25 Saudi riyals, while 786 (61.70%) traveled for less than 30 minutes to reach the facility, with most respondents (n=1009, 79.20%) owning their cars. The polymerase chain reaction (PCR) tests confirmed that 154 participants (12.09%) of the study population had contracted COVID-19, and 364 (28.57%) had been tested for the virus at least once. Nearly half of the population (n=564 (51.33%) expressed fear of visiting healthcare facilities due to COVID-19, while 308 (24.18%) were concerned about the potential risks of their age or health conditions. However, 729 (57.22%) stated that the pandemic had not affected their access to medical treatment.

Among the study participants, 411 (32.26%) reported having remote appointments; of them, 184 (44.77%) were highly satisfied, and 127 (30.90%) were satisfied. In addition, 198 (48.18%) stated they would continue with remote appointments in the future. A total of 202 participants (49.15%) had remote appointments via telephone or cellphone. The results showed that 175 participants (42.58%) stated a preference for remote appointments due to time-saving benefits.

Logistic regression analysis revealed that females were more satisfied with virtual clinics than males (OR=1.18, 95% CI (1.01, 1.40), p=0.04). The age group of 18 to 30 expressed greater satisfaction with virtual clinics compared to other age groups (OR=53.23, 95% CI (2.01, 1347.18), p=0.02). No significant associations were found among the other parameters (Table 2). See Appendix A for the full questionnaire.

**Table 2:** Logistic regression analysis on patient socio-demographic characteristics with satisfaction towards virtual clinics A p-value less than 0.05 was considered statistically significant.

Characteristics	Satisfaction with virtual clinics		p-value
	Odds ratio	95% confidence interval	
Age groups			
18 – 30	53.23	2.01, 1347.18	0.021
31 – 40	13.66	0.35, 38.26	0.277
41 – 50	15.72	0.47, 68.49	0.168
51 – 60	14.32	0.35, 53.11	0.252
61 – 70	8.23	0.10, 13.18	0.328
> 70	ref		
Gender			
Female	1.18	1.01, 1.40	0.041
Male	ref		
Education			
Below college	.918	0.27, 3.02	0.888
College	1.24	0.43, 3.58	0.686
Postgraduate	ref		
Healthcare facility			
Government hospitals	1.01	0.51, 2.03	0.956
Private hospitals	0.57	0.25, 1.29	0.181
Primary health center	0.66	0.29, 1.49	0.318
Private polyclinics	ref		
Clinic			
Dental clinic	0.94	0.06, 13.99	0.637
Diabetes and endocrine clinic	16.12	0.09, 3.45	0.965
ENT clinic	2.88	0.24, 33.9	0.251
Family medicine clinic	0.44	0.13, 1.45	0.397
Dermatology centers	0.56	0.13, 1.38	0.180
Dental clinic	0.65	0.11, 3.78	0.422
Diabetes and endocrine clinic	ref		

## Discussion

The COVID-19 pandemic resulted in significant changes in healthcare delivery worldwide, with virtual clinics playing an increasingly important role. According to the Centers for Disease Prevention and Control (CDC), telemedicine care has positively impacted access to health services during the pandemic [14]. In Saudi Arabia, the 2030 vision released in 2017 has paved the way for technology transformation.

Research has shown a disparity in access to primary healthcare services (PHCS) between urban and rural areas globally, with the rural population having the poorest access to and utilization of PHCS [15,16]. Similarly, in Saudi Arabia, the rural population is the most disadvantaged group within the population [17]. Challenges facing the healthcare system include the absence of a national crisis management policy, the lack of a national health information system, and the underutilization of the potential of electronic health strategies [15]. These challenges have resulted in low healthcare facility visits among the population, highlighting the importance of virtual clinics in Saudi Arabia.

Our study found that 564 (44.27%) participants feared visiting healthcare facilities due to COVID-19. The patients stated that their age or other health conditions might increase their health risks during their visits to healthcare facilities during the pandemic. In a previous study by Jeffery et al. conducted in Colorado, Connecticut, Massachusetts, New York, and North Carolina in the USA, noted that emergency department visits were reduced by 41.5% in Colorado and 63.5% in New York, with the most rapid reductions occurring in early March 2020 [18]. By June 2020, 41% of patients in the United States had not received their medical care, including emergency care (12%) and routine care (32%), due to COVID-19 according to Czeisler et al. [19].

The current study found that 411 participants (32.26%) had remote appointments with healthcare providers, and 311 (75.67%) of them were satisfied or highly satisfied. Among remote appointment users, 175 (42.58%) preferred them to save time. Patients frequently cite advantages such as reduced costs, fewer travel issues, and minimal time off work [20]. Mandatory social distancing and the insufficiency of effective treatments during the pandemic have made virtual services the safest interactive system between patients, both infected and uninfected, and clinicians [21].

A recent study by Alharbi et al. found that 68% of patients were satisfied with virtual services during the pandemic in Saudi Arabia [22]. The age groups 18 to 39 and 40 to 59 years were more satisfied than others, and 50.2% of males found telemedicine very convenient [22]. Another study conducted in Saudi Arabia by Thirunavukkarasu et al. reported that 54.7% of participants were highly satisfied with their telemedicine services [23]. Similarly, 52% of participants were highly satisfied with telemedicine services in another cross-sectional study by Nasser et al., which was conducted in Saudi Arabia during the pandemic [24]. Higher levels of satisfaction with virtual services in Saudi Arabia were due to internal and external factors. External factors include well-trained healthcare workers, government strategies toward virtual services, and powerful network coverage. The internal circumstances include education, socioeconomics, and innovation adoption [25-27]. Moreover, the Saudi Commission for Health Specialties consistently provides training programs for all healthcare professionals to establish telemedicine care at a global level.

Our study found that 202 (49.15%) participants had remote appointments with healthcare providers via telephone or cell phone. Prior research has demonstrated the impact of digital technologies on care pathways and service delivery in primary and secondary care [28,29]. In a previous study by Salisbury et al., most patients who underwent virtual clinics chose a phone call over a video call. Despite lower patient satisfaction, phone calls were found to be as clinically effective as usual care [30]. In an interview study with adults from the PhysioDirect telephone and advice service, participants reported using telephone services broadly but labeled them as "impersonal" and questioned their ability to achieve session goals [31].

Our study found that 198 respondents (48.18%) would continue to use virtual clinics after the pandemic. A previous study by Grossman et al. expected a decrease in virtual clinic use after the pandemic [32], while other studies have reported a higher percentage of participants desiring to continue using virtual clinics post-COVID-19. Thirunavukkarasu et al. found that 74% of participants wanted to continue using telemedicine after the pandemic [23], while Nasser et al. reported that 49% of patients preferred telemedicine post-COVID-19 [24].

As the consequences of the pandemic are expected to persist, virtual services that overcome barriers to medical treatment due to physical distancing procedures are likely to continue even after the pandemic has passed. It is necessary to prepare and encourage healthcare workers and implement government plans that deliver adequate healthcare services worldwide [33,34]. A recent review by Rajkumar et al. concluded that remote consultation has a greater impact on referral numbers, and ideally, general practitioners should be able to access a range of specialists to assist in treating various medical conditions. However, implementing such a service and maintaining high quality would likely be more efficient by incorporating it into an existing system accessible by general practices in a specific geographical area [35].

Our study found that females were more satisfied with virtual clinics than males. A previous study by Polinski et al. reported that the female gender was a predictor of liking telehealth, being very satisfied with it, and understanding virtual services [36]. Participants aged 18 to 30 years were more satisfied than other age groups, possibly due to their familiarity with technology. Similarly, Nasser et al. found that patients aged 18 to 25 years had a significantly higher mean satisfaction score compared to other participants [24].

Our study has some limitations, including online data collection, limited demographic variables analyzed, and possible response bias. We used a non-probability consecutive sampling method, and thus, limitations related to this method apply.

## Conclusions

The COVID-19 pandemic has highlighted the need to employ virtual services widely. Our study, the largest survey of patient attitudes towards attending virtual clinics during and after COVID-19, suggests a need to increase patient awareness and knowledge about virtual clinics. The majority of the participants who used virtual clinics were satisfied with the service. Nearly half of the participants wanted to continue using virtual services even after the end of the COVID-19 pandemic. Further studies assessing physicians' perceptions of virtual appointments should be encouraged.

## Appendices

Appendix A 

**Table 3:** The questionnaire distributed participants in our study SAR: Saudi riyal, MOH: Ministry of health

Demographic questions
What is your age group?
18 – 30
31 – 40
41 – 50
51 – 60
61 – 70
> 70
What is your gender?
Male
Female
What is your level of education?
Below college
College
Postgraduate
What is your nationality?
Saudi
Non-Saudi
Where are you working?
Not working
Private sector
Government sector
Retired
Others
Questions on type of hospital and clinic
What type of healthcare facility do you use?
Government hospitals
Private hospitals
MOH primary health center
Private polyclinics
Why do you visit your primary healthcare facility?
For regular check-ups
Refill my medication
Only if I have a problem
Others
Which primary clinic do you visit? Choose all that apply
Dental clinic
Diabetes and endocrine clinic
ENT clinic
Family medicine clinic
Dermatology centers
How often do you visit the healthcare facility annually?
1 – 3
4 – 6
7 – 10
>10
Do you have to travel to get to your healthcare facility?
Yes
No
Do you usually have a companion when visiting the clinics?
Yes
No
What is the cost of your trip to the healthcare facility (including fuel, parking, taxi, etc.)?
Less than 25 SAR
26 – 50 SAR
>50 SAR
How long does your trip take to the healthcare facility take?
Less than 30 minutes
31 – 60 minutes
>60 minutes
How do you go to your healthcare facility?
By my own car
By a taxi
Friend or a relative
Others
COVID-19 questions
Have you been tested for COVID-19?
Yes, and it was positive (infected)
Yes, and it was negative (not infected)
Yes, and the results were sometimes positive and sometimes negative
No
How many times have you been tested for COVID-19?
1
2
3
4
>5
Do you fear visiting the healthcare facility during the COVID-19 pandemic?
Yes
No
Do you feel that your age or any other health conditions may increase your health risks during your visit to the healthcare facility?
Yes
No
Do you take precautions during your visit to the healthcare facility?
Yes
No
Has the current pandemic affected your access to medical treatment?
No, everything is running as usual
I decided to cancel/postpone my appointments
The healthcare facility canceled/postponed my appointments
Questions on virtual clinics
Have you had any appointments related to your disease with your healthcare provider remotely since the beginning of the COVID-19 pandemic?
Yes
No
If you have had a remote appointment with your healthcare provider since the start of the pandemic, how satisfied are you with the provided service?
Highly satisfied
Satisfied
Neither satisfied nor dissatisfied
Dissatisfied
Highly dissatisfied
If you have had a remote appointment with your healthcare provider since the start of the pandemic, would you like to continue the same way?
Yes
No
How was the remote appointment conducted with your healthcare provider?
Telephone, cellphone
Through social networking applications (WhatsApp, etc. )
Video calls (Skype/Zoom audiovisual)
MOH phone services via 937
Others
How likely are you to consider remote appointments instead of in-person appointments after the pandemic?
Very likely
Likely
Neither likely nor unlikely
Unlikely
Very unlikely
Why do you prefer remote appointments?
I found out that the remote medical service is better for me
Saves my time
Less costly
More convenient than my regular visits
To avoid the traffic
